# 1953. Impact of Baseline Nutritional Status on Tuberculosis Severity in India: a multicenter prospective cohort analysis

**DOI:** 10.1093/ofid/ofad500.107

**Published:** 2023-11-27

**Authors:** Xinyi Du, Chinnaiyan Ponnuraja, Nikhil Gupte, Sonali Sarkar, Amita Gupta, Devasahayam J Christopher, Hardy Kornfeld, Vijay Viswanathan, Jerrold Ellner, C Robert Horsburgh, Chandrasekaran Padmapriyadarsini, Pranay Sinha

**Affiliations:** Boston Medical Center, Boston, MA; Indian Council of Medical Research, National Institute for Research in Tuberculosis, Chennai, Tamil Nadu, India; Johns Hopkins University, Pune, Maharashtra, India; Jawaharlal Institute of Postgraduate Medical Education and Research, Puducherry, Puducherry, India; Johns Hopkins, Baltimore, MD; Christian Medical College, Vellore, Ranipet district, Tamil Nadu, India; Department of Medicine, University of Massachusetts Chan Medical School, Worcester, Massachusetts; Prof. M. Viswanathan Diabetes Research Centre, Chennai, Tamil Nadu, India; Center for Emerging Pathogens, Department of Medicine, New Jersey Medical School, Rutgers Biomedical and Health Sciences, Newark, New Jersey; Boston University, Boston, Massachusetts; Indian Council of Medical Research, National Institute for Research in Tuberculosis, Chennai, Tamil Nadu, India; Boston University, Boston, Massachusetts

## Abstract

**Background:**

India bears a quarter of the global tuberculosis (TB) burden. Previous studies have shown that more than 50% of persons with TB in India are undernourished and that undernutrition is associated with increased risk of unfavorable TB outcomes and mortality. The effect of nutritional status may be explained by its effect on other factors associated with poor outcomes. In this study, we assessed the impact of undernutrition at TB detection on markers of TB severity.

**Figure:**
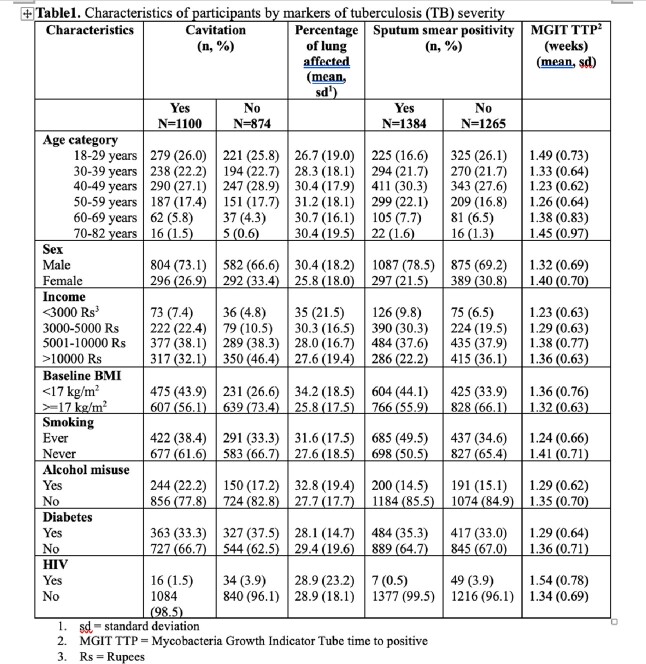

**Methods:**

We analyzed prospectively collected data for persons with TB aged ≥ 18 years from five Regional Prospective Observational Research for Tuberculosis (RePORT) - India sites. We built multivariable regression models to assess relationships between undernutrition at TB detection (baseline body mass index (BMI), kg/m^2^) and markers of severity. We used logistic regression for lung cavitation and high-grade sputum smear positivity (defined as smear grade ≥ 2+), and linear regression for percentage of lung affected and time to positivity of Mycobacteria Growth Indicator Tube (MGIT; weeks). The models included age, sex, symptom duration a priori and all variables with p< 0.2 in univariate analysis.

**Results:**

We had baseline BMI on 2898 persons with TB and 1132 (39.06%) of participants had moderate-severe undernutrition (BMI< 17kg/m^2^) at treatment initiation. After adjusting for age, sex, symptom duration, income, HIV status, diabetes, smoking, and alcohol misuse (defined as having an AUDIT-c score ≥ 3 for females and ≥ 4 for males), baseline BMI< 17kg/m^2^ was associated with increased odds of lung cavitation (aOR 1.67, 95% confidence interval [CI]: 1.32, 2.10), increased odds of high bacillary sputum grade (aOR 1.33, 95%CI: 1.12, 1.60), an average 7.13% (95%CI: 5.11, 9.16) more lung affected, and an average 0.045 (95%CI: -0.024, 0.11) longer time to liquid culture positivity.

**Figure:**
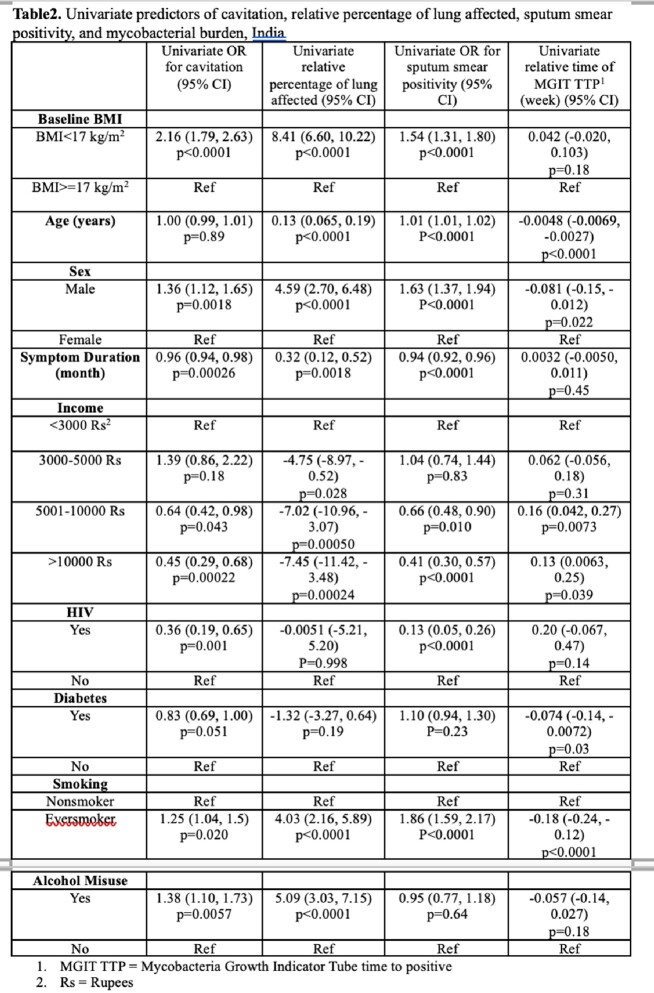

**Figure:**
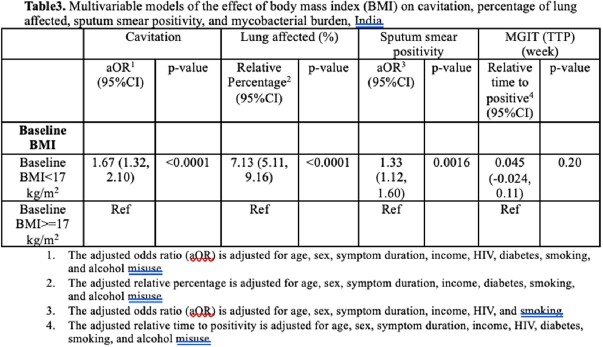

**Conclusion:**

To our knowledge, this is the largest prospective cohort study evaluating the association between undernutrition and markers of TB severity. While our analysis cannot account for the bidirectional relationship between TB disease and nutritional status, it does highlight the impact of systematic nutritional assessment in persons with TB.

**Disclosures:**

**Devasahayam J. Christopher, DNB, FRCP**, Abbot, India: Honoraria|Astra Zeneca: Honoraria|Cipla, India: Honoraria|JB Chemicals: Honoraria|Lupin pharmaceuticals: Honoraria|Pfizer: Honoraria|Zydus Cadila: Honoraria

